# LC-MS/MS Validation Analysis of Trastuzumab Using dSIL Approach for Evaluating Pharmacokinetics

**DOI:** 10.3390/molecules21111464

**Published:** 2016-11-02

**Authors:** Rohit H. Budhraja, Milin A. Shah, Mahendra Suthar, Arun Yadav, Sahil P. Shah, Prashant Kale, Parisa Asvadi, Mariadhas Valan Arasu, Naif Abdullah Al-Dhabi, Chun Geon Park, Young-Ock Kim, Hak Jae Kim, Y. K. Agrawal, Ravi. K. Krovidi

**Affiliations:** 1 Panomics Lambda Therapeutic Research Limited, Gota, Ahmedabad, Gujarat 382481, India; rohitbudhraja@gmail.com (R.H.B.); milinshah@lambda-cro.com (M.A.S.); mahendramevada8@gmail.com (M.S.); arunyadavpoo@gmail.com (A.Y.); sahilshah.ind@gmail.com (S.P.S.); prashant@lambda-cro.com (P.K.); 2 Department of Pharmaceutical Sciences, Gujarat Forensic Sciences University, Gandhinagar 382481, India; drykagrawal@yahoo.com; 3 Analytical Development and Global Regulatory Affairs, Intas Pharmaceuticals Ltd., Ahmedabad, Gujarat 382213, India; parisa_asvadi@intaspharma.com; 4 Department of Botany and Microbiology, Addiriyah Chair for Environmental Studies, College of Science, King Saud University, P.O. Box 2455, Riyadh 11451, Saudi Arabia; mvalanarasu@gmail.com (M.V.A.); naldhabi@ksu.edu.sa (N.A.A.-D.); 5 Department of Medicinal Crop Research, Rural Development Administration, Eumseong, Chungbuk 369-873, Korea; pcg@korea.kr; 6 Department of Clinical Pharmacology, College of Medicine, Soonchunhyang University, Cheonan 31151, Korea

**Keywords:** trastuzumab, LC-MS/MS, dSIL, pharmacokinetics

## Abstract

Quantitative targeted proteomics based approaches deploy state-of-the-art Liquid chromatography tandem mass spectrometry LC-MS technologies and are evolving as a complementary technique to standard ligand-binding based assays. Advancements in MS technology, which have augmented the specificity, selectivity and sensitivity limits of detection and freedom from antibody generation, have made it amicable towards various clinical applications. In our current work, a surrogate peptide based quantitative proteomics assessment is performed by selecting specific signature peptides from the complementary determining region CDR region of trastuzumab (Herclon^®^, Roche products in India). We developed a double Stable Isotope Label (dSIL) approach by using two different surrogate peptides to evaluate the proteolytic digestion efficiency and accurate quantification of the target analyte peptide of Herclon^®^ in human serum. Method validation experiments were meticulously performed as per bioanalytical method validation guidelines. The dSIL approach, using an LC-MS/MS based quantification assay demonstrated good linearity over a range of 5–500 µg/mL of Herclon^®^, and validation experimental data is in compliance with bioanalytical regulatory guidelines.

## 1. Introduction

Trastuzumab, a humanized monoclonal antibody, is widely used for the treatment of various cancers in humans including metastatic breast cancer and gastric cancer with over expression of cell surface human epidermal growth factor receptor 2 (HER2) receptors. This over-expression of HER2 receptors leads to abnormal cellular signaling, and is responsible for the abnormal proliferation of cells resulting in malignancy. These receptors are activated upon ligand binding and form homodimers or heterodimers. Dimerization results in auto-phosphorylation and/or trans-phosphorylation of specific tyrosine residues in the intracellular domains of HER2. A cascade of downstream signaling events, in turn, leads to the activation of the Ras/Raf/mitogen-activated protein kinase, the phosphoinositide 3-kinase/Akt, and protein kinase C (PKC) pathways [1,2].

Trastuzumab is a mediator of antibody-dependent cellular cytotoxicity (ADCC), it inhibits the dimerization of the HER2 receptor, and is thereby primarily used in HER2 positive breast cancer treatment [3,4,5]. The US Food and Drug Administration (USFDA) approved trastuzumab (HERCLON™, Roche products in India) in 1998 for the treatment of human metastatic breast cancer [6]. Trastuzumab is administered by intravenous infusions of 10 to 500 mg once every week with an initial dose of 8 mg/kg infused over 90 min, followed by 6 mg/kg over 30 to 90 min every 3 weeks. The compound has demonstrated dose-dependent pharmacokinetics, with an average half-life of two and 12 days at the 10- and 500-mg dose levels, respectively [7,8]. At the highest weekly dose studied (500 mg), mean peak serum concentrations were 377 μg/mL [9].

Ligand-binding assays (LBA) serve as a gold standard for evaluating the quantitative levels of biomolecules in complex human matrices. However, limitations in reagent specificity, interferences from anti-drug antibodies, cross reactivity related issues, and antibody related long method development time impede the utilization of this approach [9,10]. While ligand-binding assays (LBAs) have historically been the only platform available for protein bioanalysis, Liquid Chromatography-Mass Spectrometry LC-MS based technology is now providing an alternative approach which complements LBAs. The LC-MS/MS method is rapidly evolving as a complementary tool for PK assessments [11,12].

The surrogate peptide approach is most widely used for the quantification of protein biotherapeutics including monoclonal antibodies (mAbs). It exploits the unique selectivity of triple quadrupole MS in differentiating the isotopes of the same element of the endogenous peptides and surrogate labeled peptides; they possess intrinsic physical and chemical properties and separations are identical on the chromatographic scale, correcting the variability and resulting in an enhanced precision. Reliable quantification is achieved by MS ionization and enables the calculation of absolute abundance. This approach mitigates the risks associated with background signal interferences resulting from complex biological matrices. The surrogate peptide approach offers highly reliable and accurate quantification of large biomolecules without the need of developing any specific antibodies.

A method of absolute quantitation that is based on multiple reaction monitoring (MRM) is performed by spiking stable isotope-labeled (SIL) synthetic peptide in a complex biological matrix and serves as an internal standard for accurate quantification. The synthesis of the SIL peptides is cost-effective and the associated shorter LC-MS method development time makes it a method of choice. Targeted proteomics deploys state-of-the art LC/MS technology, provides a robust approach for the specific detection and quantification of the target signature peptides from complex biological matrices. The use of targeted proteomics in recent studies has been demonstrated for the quantification of trastuzumab in human plasma [13] and rat plasma [14] using single reaction monitoring (SRM) mode LC-MS/MS and LC-TOF (Time-of-flight)- MS/MS respectively. In our current study, we have used this approach to evaluate the pharmacokinetic study of trastuzuamb in human serum with a more reliable and robust approach. The quantitative targeted proteomics approach maintains an appropriate sampling rate and limits the number of analytes measured in one experiment. The triple quadrupole (QQQ) MS has two stages of mass filtering in Q1 and Q3, while collision induced dissociation (CID) occurs in Q2. Selective transitions of the precursor ion are analyzed in Q3. Skyline is a publicly available open resource tool; it simplifies the development of the MRM method for targeted proteomics approach [15]. It generates a transition list containing the information of peptides with their precursor and fragment ions. It provides the declustering potential and collision energy levels for MRM transitions enabling rapid MS method development.

Trypsin enzyme is a serine protease, which cleaves at the carboxyl terminal of lysine/arginine residues and is widely used in proteomics studies. Efficient proteolytic digestion is critical towards generating targeted peptides needed for accurate quantification. Partial digestion could potentially result in non-availability of signature peptides required for accurate quantification. However, in 2004 Bamidge et al. demonstrated the incorporation of tryptic cleavage side in synthetic internal standard in protein quantification. We have developed an approach by using two different internal standard synthetic peptides; one with an extended tryptic cleavable site and a second without a cleavable site. The dSIL approach involves two internal labeled surrogate peptide standards; for performing an accurate quantification and for critically monitoring the tryptic digestion efficiency. Protein identifications resulting from two unique peptides provide a higher level of confidence and are adopted in our current approach.

In our current study, we have developed a robust dSIL surrogate peptide based approach for an accurate and reliable quantification of mAb Herclon^®^ (Fedelty Health Care Private Limited, Mumbai, India) spiked in human serum with the LC-MS/MS approach using the Skyline and MRM (multiple reaction monitoring) methodology was developed for assessing the digestion efficiency and monitoring the PK endpoints ranging from 5 µg/mL to 500 µg/mL. Linearity R^2^ = 0.99 was achieved; the developed method is highly reproducible and is potentially applicable for clinical studies to evaluate the dosage response of a biosimilar Trastuzumab in human serum.

## 2. Results and Discussion

### 2.1. MRM Transitions

The precision and accuracy results (Table 1, Table 2 and Table 3) of selected signature peptide MRM transitions (Supplementary Tables S1 and S2) demonstrate a robust and reproducible method delivering a high quality dataset for the accurate quantification of trastuzumab in human serum. We evaluated the repeatability, reproducibility and robustness of sample preparation under controlled assay conditions, and the consistency of LC separation by performing three constitutive precision and accuracy (P&A) batches of Trastuzumab. The computed intra-assay precision yielded a percentage of Coefficient of Variability (CV) of 6.4%, 10.1%, 9.2%, 9.3% respectively, whereas the intra-assay accuracy; 101.9%, 99.6%, 97.3%, 104.2% was observed for Lower-limit of quantitation (LOQ-QC), Low-quality control (LQC), medium quality control (MQC) and high quality control (HQC) respectively. Our results comply with the bioanalytical regulatory guideline requirements and are well under acceptable limits. Our quantitative data suggests the developed dSIL method is capable of a precise and accurate measurement of trastuzumab in human serum to a minimal level with a LLOQ reaching up to 5 µg/mL (Figure S1).

The surrogate peptide containing a flanking region (FTISADTSK) was also quantified in three different P&A batches for monitoring the tryptic digestion efficiency of trastuzumab spiked human serum. Results obtained from each point of linearity have demonstrated a good accuracy (Table 1) confirming a robust and reliable tryptic digestion of serum samples. Additional surrogate peptide (IYPTNGYTR) spiked into the serum followed by MS quantification (Table 2) was also evaluated and yielded highly reliable, reproducible and accurate quantification of the target analyte.

### 2.2. Selection of Signature Peptides

Selected MRM transitions were acquired and the resulting raw files were analyzed using MultiQuant software for comparing the peak area responses of various tryptic peptide transitions. Meticulous manual data analysis was performed to filter the peptides resulting from the Fc region as the fragment crystallisable (Fc) region peptides show a high degree of sequence similarity to endogenous Immunoglobulin G (IgG) in the human serum. Peptide sequences with charge states +2; FTISADTSK (504.772/667.341) and IYPTNGYTR (542.775/808.395) yielded a higher MS response, signature peptides were further confirmed to be located in the fragment antigen-binding (Fab) region by pepsin digestion (data not shown) and uniquely represent the CDR regions of trastuzumab. We have performed protein BLAST search for the selected peptides and the negative result of BLAST for both peptides must probably indicate the presence of both peptides in the CDR region. The LC-MS/MS acquisition parameters were optimized to ensure discrete separation on the LC time scale devoid of any specific background interference from the complex serum biological matrix.

### 2.3. Validation Experiments

#### 2.3.1. Precision and Accuracy

The precision and accuracy results (Table 1, Table 2 and Table 3) of selected signature peptide MRM transitions (Tables S1 and S2) demonstrate a robust and reproducible method delivering a high quality dataset for accurate quantification of trastuzumab in human serum. We evaluated the repeatability, reproducibility and robustness of sample preparation under controlled assay conditions, and the consistency of LC separation by performing three constitutive P&A batches of Trastuzumab. The computed intra-assay precision yielded a percentage of CV of 6.4%, 10.1%, 9.2%, 9.3% respectively, whereas for the intra-assay accuracy, 101.9%, 99.6%, 97.3%, 104.2% was observed for LOQ-QC, LQC, MQC and HQC respectively. Our results comply with the bioanalytical regulatory guideline requirements and are well under acceptable limits. Our quantitative data suggests the developed dSIL method is capable of a precise and accurate measurement of trastuzumab in human serum to a minimal level with a LLOQ reaching up to 5 µg/mL (Figure S1).

The surrogate peptide containing a flanking region (FTISADTSK) was also quantified in three different P&A batches for monitoring the tryptic digestion efficiency of trastuzumab spiked human serum. Results obtained from each point of linearity have demonstrated a good accuracy (Table 1) confirming a robust and reliable tryptic digestion of serum samples. Additional surrogate peptide (IYPTNGYTR) spiked into the serum followed by MS quantification (Table 2) was also evaluated and yielded highly reliable, reproducible and accurate quantification of the target analyte.

#### 2.3.2. Stability

Monoclonal antibodies are complex biological molecules and their stability assessments need to be performed for evaluating the intactness during analytical assessments, sample storage and processing conditions. Stability assessment of trastuzumab in human serum is evaluated by performing multiple freeze-thaw cycles at −65 ± 10 °C and for 6 h storage at +4 °C. The human serum containing trastuzumab was subjected to four consecutive freeze–thaw cycles across two different concentrations including low QC and high QC. We additionally evaluated low QC and high QC trastuzumab spiked serum sample stored for 6 h at +4 °C and performed tryptic digestion experiments and compared with freshly prepared low QC and high QC samples (Table 4 and Table 5). Results from stability samples and control samples showed a high degree of overlap and comply with the regulatory requirements, indicating higher stability conditions of trastuzmab. We additionally evaluated the intact stability by direct infusion studies using QTOF-MS (data not shown).

Whole blood stability was verified using six immediately prepared different HQC samples and LQC samples, level in whole blood for stability samples and compared with freshly spiked HQC and LOQC samples. The percent mean ratio of the peak area ratio for the low (97.2%) and high (100.1%) quality control sample was within the acceptance range of 85%–115% while comparing the result with freshly spiked quality control samples.

An auto sampler stability test was performed by storing the tryptic digest mixture in the LC auto sampler for 96 h maintained at +4 °C. MRM acquisition of the auto sampler stability test samples and control samples demonstrated similar MS response as expected; peptides are inherently stable and are much less prone to any further degradation due to temperature or storage conditions.

#### 2.3.3. Selectivity and Matrix Effect

Selectivity of the method was tested by processing serum collected from a total of ten different human subjects; including six different human sera, two hemolyzed and two lipemic serum samples. Trastuzumab and Internal standard (ISTD) were spiked in only one lot of sera at a LLOQ level of 5 µg/mL. No significant interference was observed and the average blank area response was 1.5% when compared to the area response of LLOQ which was found within the acceptance criteria (≤30%). The area observed at the retention time of ISTD in blank was around 0.004% as compared to the area response of the LLOQ sample, which was found within the acceptance criteria (≤5%). Our results are highly reproducible. Results from the current study indicate a high degree of selectivity and the method is robust and is in concordance with the regulatory guidelines.

The matrix effect determines whether co-eluting matrix components modify the detector response. The matrix effect is evaluated by analyzing six lots of serum along with two hemolyzed and two lipemic serums fortified with analyte at the concentration of low and high QC. Four aliquots of each low and high QCs of each matrix were analyzed using freshly processed calibration curve samples. Precision of low (6.7%) and high (7.2%) quality control samples was within the acceptance criteria of ≤20%. Accuracy of low (83.9%) and high (101.5%) quality control samples was within acceptance criteria of 80%–120% of the nominal. The above experiment indicates the negligible effect of matrix on the analysis of trastuzumab and ISTD.

#### 2.3.4. Auto Sampler Carryover

We evaluated the auto sampler carryover to ensure and monitor carryover contamination. Sequential runs of blank and ULOQ were sequenced for analysis and terminally analysed LLOQ. Results indicate a complete carryover free autosampler condition, blank runs acquired post ULOQ injections did not indicate any considerable signal and were at exceedingly lower levels, below <5% LLOQ response and in fair agreement with the regulatory guidelines. The auto sampler carryover was monitored throughout the study.

## 3. Materials and Methods

### 3.1. Chemicals and Materials

Trastuzumab standard (Herclon) with a concentration of 440 mg from Roche, India was used in this study. LC-MS grade acetonitrile, water, methanol from Fisher Scientific (Loughborough, UK); formic acid and ammonium bicarbonate from Fluka (St. Louis, MO, USA); Rapigest solution from Waters (Milford, MA, USA); dithiothreitol, and iodoacetamide from Sigma (St. Louis, MO, USA); sequencing grade modified trypsin from Worthington, Lakewood, NJ, USA were procured. Internal standard (ISTD) of trastuzumab signature peptides with a heavy labeled (^13^C^15^N-isotopically-KRFTISADTSK) was synthesized by Thermo (Waltham, MA, USA) and IYPTNGYTR was synthesized by commercial supplier, JPT peptide Inc. (Berlin, Germany).

### 3.2. Standard Solution

Stock solutions of trastuzumab (Herclon, Roche, India) were prepared at 21 mg/mL in bacteriostatic water for injection (BWFI) as per the manufacturer’s instructions and were stored at +4 °C until needed. Intermediate spiking solutions (2.1 mg/mL) were prepared using freshly processed human serum. Intermediate solution was serially diluted with blank serum to prepare standards (5, 10, 25, 75, 250, 375, 450, 500 µg/mL) and QC samples (5, 15, 250 and 396 µg/mL).

Pre-weighed trastuzumab internal standard (ISTD) (equivalent to about 1.0 mg) was dissolved in LC/MS grade water to produce a stock solution of 1 mg/mL and the stock solution was further diluted using LC/MS grade water to achieve a final concentration 2.5 µg/mL ISTD dilution.

All CC and QC samples and ISTD solutions were prepared on ice and stored at – 65 ± 10 °C until needed.

### 3.3. Generation of Transition List

Skyline was used to generate a predicted MRM transition list containing tryptic peptides of trastuzumab for subsequent multiple reaction monitoring (SRM/MRM) analysis. Skyline parameters include tryptic cleavage; mass range of 50–1250 *m*/*z* for all tryptic peptides; QTRAP SCIEX MS in high resolution *m*/*z* mode setting.

### 3.4. Sample Preparation (Double SIL-dSIL Approach)

Serum samples (20 µL) were processed for evaluating digestion efficiency and accurate quantification by using sequential SIL peptides (^13^C^15^N-isotopically labeled peptide) spiking GRFTISADTSK and IYPTNGYTR, respectively. In-solution tryptic digestion was performed as mentioned in the information dependent data acquisition (IDA) analysis section and additionally an extended stable isotope labeled (SIL) peptide (the ^13^C^15^N-isotopically labeled extended peptide GRFTISADTSK) was added (25 µL) from stock vial (2500 ng/mL) to each sample tube and they were vortexed well. Samples were denatured using 0.1% Rapigest solution and incubated at 80 °C for 10 min. Reduction was performed using DTT (Dithiothreitol) solution having a final concentration of 20 mM at 60 °C for 45 min. It was further alkylated using IAM (Iodoacetamide) with the final concentration at 20 mM and incubated at room temperature in the dark. Sequencing grade modified trypsin was added to the samples and incubated overnight at 37 °C. The tryptic digest serum sample mixture was quenched using 1% formic acid.

Acidified tryptic peptide mixture was desalted using Waters mix-mode cation exchange (MCX) Oasis cartridges (30 mg/1 mL). Cartridges were conditioned with 1.0 mL methanol [HPLC Grade] and 1.0 mL of 0.1% formic acid solution on solid-phase extraction (SPE) manifold by applying low pressure. Acidified tryptic digest mixture was loaded on to the cartridges, and washed with 1.0 mL of 0.1% formic acid solution (*v*/*v*), peptides were finally eluted using 250 µL (2×) of elution mixture; 2% ammonium hydroxide in acetonitrile; and water (50:50) into pre-labeled collection tubes. The eluate was speed-vac concentrated at ambient temperature. Dried tryptic peptide mixture was re-constituted in 200 μL of 0.1% formic acid in 5% acetonitrile for LC-MS/MS analysis.

### 3.5. LC-MRM Method Conditions

Tryptic peptide mixture was separated using a reverse phase LC separation with Acquity column (150 mm × 2.1 mm) packed with Ethylene Bridged Hybrid (BEH) C18 1.7 μm sized resin. A sample volume of one µL was injected using Shimadzu Nexera ultra-high performance liquid chromatography (UHPLC) (Shimadzu, Japan) coupled with Q-trap SCIEX 6500 (QQQ) mass spectrometer (SCIEX). The column oven temperature was maintained at 40 °C, with a flow rate of 0.3 mL/min. A linear gradient was applied for 12 min ramping from 3% to 85% solvent B (0.1% formic acid in 95% acetonitrile).

The following MS parameters were applied for data acquisition using a SCIEX 6500 triple quadrupole system. Positive electrospray; ion source voltage at 5500 V; source temperature at 550 °C; Collision activate dissociation (CAD) at 12; Nebulizer gas (GS1) at 40; and Auxiliary gas (GS2) at 60. A Skyline predicted transition list with a dwell time of 200 ms was included for LC-MS/MS MRM acquisition.

## 4. Conclusions

This current study demonstrates the strength of the dSIL approach for the quantitative evaluation of mAb biologics in human biological matrix. An LC-MS/MS based targeted proteomics approach was applied for method development and method validation towards the evaluation of the pharmacokinetic study of trastuzumab in human serum. We have successfully developed the dSIL (double stable isotope labeled) method for a precise and accurate quantification of trastuzumab ranging from 5 µg/mL to 500 µg/mL. Validation experiments including precision and accuracy batches, cross contamination, auto sampler carryover, selectivity were performed as per regulatory guidelines. Validation experiments met the regulatory acceptance criteria, indicating the validation acceptability of the established method. The developed dSIL approach might be useful for the evaluation of the pharmacokinetic study of trastuzumab in clinical human serum samples. We are currently extending the method for the evaluation of a clinical study required for regulatory submission.

## Figures and Tables

**Table 1:** Table represents the quantification data of trastuzumab signature peptide (IYPTNGYTR) in human serum batch; calibration curve (CC) extends from 5 µg/mL to 500 µg/mL; their corresponding accuracy percentage and R^2^ = 0.99 was observed.

Sample Name (CC)	Analyte Peak Area	Analyte Retention Time (RT) (min)	Internal Standard (IS) Peak Area	IS RT (min)	Area Ratio	Analyte Conc (µg/mL)	Calculated Conc (µg/mL)	Accuracy (%)
STD_8	2201571	4.476	1269609	4.471	1.734	501.6	526.911	105
STD_7	1764292	4.563	1184001	4.557	1.49	451.44	452.73	100.3
STD_6	1523150	4.528	1249879	4.523	1.219	375.598	370.177	98.6
STD_5	1075654	4.46	1216198	4.456	0.884	251.651	268.549	106.7
STD_4	289982	4.537	1226623	4.534	0.236	75.495	71.487	94.7
STD_3	101021	4.534	1244545	4.531	0.081	25.668	24.281	94.6
STD_2	44585	4.514	1299658	4.511	0.034	10.267	10.029	97.7
STD_1	23549	4.501	1264843	4.5	0.019	5.134	5.259	102.4

**Table 2:** Quantification data of trastuzumab signature peptide (FTISADTSK) in human serum batch; calibration curve (CC) extends from 5 µg/mL to 500 µg/mL; their corresponding accuracy percentage and R^2^ = 0.99 was observed.

Sample Name (CC)	Analyte Peak Area	Analyte RT (min)	IS Peak Area	IS Retention Time (min)	Area Ratio	Analyte Conc (µg/mL)	Calculated Conc (µg/mL)	Accuracy (%)
STD_8	2826503	6.058	513894	6.056	5.500	501.600	495.319	98.7
STD_7	2668953	6.077	554130	6.075	4.816	451.440	433.648	96.1
STD_6	2174781	6.051	526182	6.048	4.133	375.598	372.009	99.0
STD_5	1201036	6.075	436818	6.074	2.750	251.651	247.200	98.2
STD_4	432522	6.081	506952	6.079	0.853	75.495	76.145	100.9
STD_3	251462	6.140	908537	6.139	0.277	25.668	24.152	94.1
STD_2	69119	6.123	521644	6.120	0.133	10.267	11.138	108.5
STD_1	44541	6.118	623461	6.116	0.071	5.134	5.630	109.7

**Table 3:** Precision and accuracy of LC-MS/MS method.

Parameters	Lower-Limit of Quantitation (LOQ QC)	Low-Quality Control (LQC)	Medium Quality Control (MQC)	High Quality Control (HQC)
Calculated concentration (µg/mL)	5.416	16.237	223.708	397.879
	5.421	16.366	252.093	462.144
	5.374	13.4	216.874	409.452
	4.857	15.199	264.181	449.311
	5.569	13.163	269.631	400.535
	5.94	16.551	229.291	356.099
Mean	5.4295	15.1527	242.6297	412.5700
Standard deviation (SD±)	0.34962	1.52552	22.28179	38.38449
Precision (CV%)	6.4	10.1	9.2	9.3
Nominal value (µg/mL)	5.326	15.218	249.480	396.000
Accuracy%	101.9	99.6	97.3	104.2
Number of samples (N)	6	6	6	6

**Table 4:** Stability data of trastuzumab signature peptide (IYPTNGYTR) after 6 h incubation at 4 °C in human serum batch. Mean ratio; 100.2% and 107.9% and % change; −5.9 and 13.9 was observed at HQC and LQC level, respectively. Here, n represents the number of samples analyzed for the stability experiment. (Acceptance criteria: %Change ≤15%; %Mean ratio 90%–110%, %CV ≤20%).

Parameters	Freshly Injected		After 6.0 h	
	HQC (µg/mL)	LQC (µg/mL)	HQC (µg/mL)	LQC (µg/mL)
**Calculated concentration of signature peptide**	371.105	16.441	348.739	18.068
	388.888	15.735	337.011	17.899
	383.461	12.462	378.322	16.052
	337.262	18.304	391.093	17.159
	381.139	15.744	414.900	17.195
	369.525	16.588	365.539	16.448
**Mean**	371.8967	15.8790	372.6007	17.1368
**SD**	18.51309	1.91906	28.45981	0.78781
**Coefficient of Variability (CV)**	5.0	12.1	7.6	4.6
**Nominal Concentration (µg/mL)**	396	15.144	396	15.144
**N**	6	6	6	6
% Change			−5.9	13.2
% of Mean ratios			100.2	107.9

**Table 5:** Table demonstrates the stability data of trastuzumab signature peptide (IYPTNGYTR) after four freeze-thaw cycles of the human serum batch. Mean ratio; 100.0% and 104.0%and %change; −6.1 and 9.1 was observed at HQC and LQC level, respectively. Here, n represents the number of sample analyzed for the stability experiment. (Acceptance criteria: %Change ≤15%; % Mean ratio 90%–110%, %CV ≤20%).

Parameters	Freshly Injected		After 4 FT CYCLE	
	HQC (µg/mL)	LQC (µg/mL)	HQC (µg/mL)	LQC (µg/mL)
**Calculated Concentration of signature peptide**	371.105	16.441	290.497	15.902
	388.888	15.735	383.686	16.895
	383.461	12.462	414.378	16.081
	337.262	18.304	408.799	17.457
	381.139	15.744	427.938	16.552
	369.525	16.588	305.989	16.244
**Mean**	371.8967	15.8790	371.8812	16.5218
**SD**	18.51309	1.91906	59.01829	0.57800
**CV**	5.0	12.1	15.9	3.5
**Nominal value (µg/mL)**	396	15.144	396	15.144
**N**	6	6	6	6
% Change			−6.1	9.1
% of Mean ratios			100.0	104.0

## Supplementary Materials

Supplementary materials can be accessed at: http://www.mdpi.com/1420-3049/21/11/1464/s1.
