# Supplementary material for: LC-MS/MS Validation Analysis of Trastuzumab Using dSIL Approach for Evaluating Pharmacokinetics
Source: Molecules. 2016 Nov 2;21(11):1464. doi: 10.3390/molecules21111464 (PMC6274275; doi:10.3390/molecules21111464)
Supplement: Supplementary file 1 [file molecules-21-01464-s001.pdf]

Molecules **2016**, *21*, 1464; doi:10.3390/molecules21111464

Supplementary Materials: LC-MS/MS Validation Analysis of Trastuzumab Using dSIL Approach for Evaluating Pharmacokinetics

Rohit H Budhraja, Milin A. Shah, Mahendra Suthar, Arun Yadav, Sahil P Shah, Prashant Kale, Parisa Asvadi, Mariadhas Valan Arasu, Naif Abdullah Al-Dhabi, Chun Geon Park, Young-Ock Kim, Hak Jae Kim, Y.K. Agrawal and Ravi. K. Krovidi

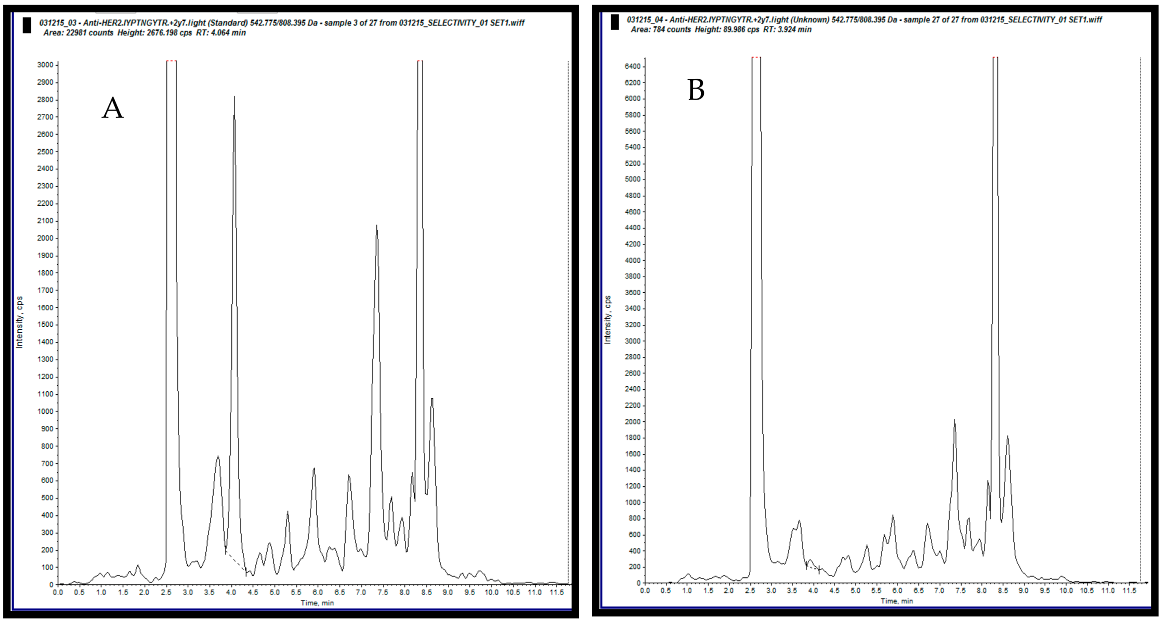

Figure S1. Extracted ion chromatograms with the transition for trastuzumab signature peptide (IYPTNGYTR) (**A**) an LLOQ of 5 µg/mL; (**B**) Extracted blank plasma.

Table S1. Data represents statistics of precision and accuracy results of analyte (Trastuzumab) of three different P&A batches.

Parameters	Trastuzumab in Human Plasma (µg/mL)<br>Quality Control Samples Id (Prepared from Biosimilar)					
	DIL QC (1/10)	HQC	MQC	LMQC	LQC	LOQ QC
P&A I	2314.674	367.602	203.047	47.413	15.013	4.875
	2324.009	359.434	193.510	50.546	15.170	4.550
	2336.139	372.557	204.702	46.737	13.746	4.954
	2324.399	381.304	207.476	49.421	14.773	4.893
	2310.723	346.703	193.750	53.974	14.586	5.347
	2361.571	385.922	201.510	55.380	14.611	5.088
Mean	2328.5858	368.9203	200.6658	50.5785	14.6498	4.9512
SD ± µg/mL	18.41830	14.42897	5.79804	3.48431	0.49802	0.26288
Precision (%CV)	0.8	3.9	2.9	6.9	3.4	5.3
Nominal value	2450.000	378.000	204.120	51.030	14.926	5.131
Accuracy (%)	95.0	97.6	98.3	99.1	98.1	96.5
n	6	6	6	6	6	6
P&A II	2148.847	346.819	186.611	42.509	15.022	5.544
	2166.138	347.109	174.232	44.819	15.623	5.270
	2060.092	368.158	194.370	51.743	15.406	5.463
	2101.318	348.566	183.374	48.979	13.462	5.297
	2122.772	344.791	188.950	48.290	13.614	5.541
	2130.259	347.527	192.679	43.640	15.416	5.233
Mean	2121.5710	350.4950	186.7027	46.6633	14.7572	5.3913
SD ±	37.40848	8.74100	7.29604	3.56685	0.96531	0.14109
Precision (%CV)	1.8	2.5	3.9	7.6	6.5	2.6
Nominal value	2450.000	378.000	204.120	51.030	14.926	5.131
Accuracy (%)	86.6	92.7	91.5	91.4	98.9	105.1
n	6	6	6	6	6	6
P&A III	2523.528	368.223	201.997	47.888	15.584	5.020
	2400.532	359.497	189.935	51.007	14.868	4.283
	2523.477	372.777	205.770	45.062	14.214	4.745
	2474.735	380.563	201.321	48.534	15.338	4.333
	2544.001	340.218	192.853	52.998	15.523	4.359
	2434.657	387.701	203.437	54.654	14.447	5.733
Mean	2483.4883	368.1632	199.2188	50.0238	14.9957	4.7455
SD ±	56.93316	16.80957	6.31762	3.53983	0.57788	0.56284
Precision (%CV)	2.3	4.6	3.2	7.1	3.9	11.9
Nominal value	2450.000	378.000	204.12000	51.03000	14.926	5.131
Accuracy (%)	101.4	97.4	97.6	98.0	100.5	92.5
n	6	6	6	6	6	6

Table S2. Data represents statistics of Matrix effect on analyte (Trastuzumab).

Parameters	Matrix	Quality Control Samples Id	
		HQC	LQC
		Back-Calculated Values (µg/mL)	
Matrix effect	Normal Serum	378.244	11.308
		400.897	12.377
		383.383	12.782
		355.713	12.530
	Normal Serum	365.054	11.750
		387.054	11.209
		434.353	12.583
		351.735	12.089
	Normal Serum	395.112	13.338
		423.647	12.341
		409.468	13.291
		380.965	13.316
	Normal Serum	359.081	11.550
		423.037	11.374
		413.693	13.164
		335.534	13.288
	Normal Serum)	339.487	10.889
		395.453	12.731
		433.538	12.843
		392.987	12.871
Matrix effect (22/03/16)	Normal Serum	332.623	12.387
		412.354	12.472
		380.048	13.009
		404.777	12.820
	Lypemic Serum	398.760	13.056
		390.322	11.748
		385.010	13.989
		397.191	14.114
	Lypemic Serum	372.180	11.756
		388.893	11.854
		309.176	12.389
		394.988	12.958
	Haemolysed Serum	365.456	11.939
		376.558	12.587
		363.404	13.711
		397.910	12.905
	Haemolysed Serum	385.830	10.166
		361.026	12.883
		371.605	13.150
		397.664	13.141
Mean		383.6053	12.5165
SD ±		27.51636	0.84293
Precision (%CV)		7.2	6.7
Nominal value (ng/mL)		378.000	14.926
Accuracy (%)		101.5	83.9
n		40	40

Note: Underlined values were not within the acceptance range (± 20% of nominal value for all QCs) but included for the calculation.
